# Five-Fraction Stereotactic Radiosurgery With Non-Contrast-Enhanced MRI-Based Target Definition and Moderate Dose Spillage Margin for Limited Brain Metastases With Impaired Renal Function

**DOI:** 10.7759/cureus.37384

**Published:** 2023-04-10

**Authors:** Kazuhiro Ohtakara, Kojiro Suzuki

**Affiliations:** 1 Department of Radiation Oncology, Kainan Hospital Aichi Prefectural Welfare Federation of Agricultural Cooperatives, Yatomi, JPN; 2 Department of Radiology, Aichi Medical University, Nagakute, JPN

**Keywords:** contrast media, gross tumor volume, t2-weighted image, renal failure, contrast enhancement, volumetric modulated arc therapy, target definition, stereotactic radiosurgery, hypofractionation, brain metastasis

## Abstract

In stereotactic radiosurgery (SRS) planning for brain metastases (BMs), the target volume is usually defined as an enhancing lesion based on contrast-enhanced (CE) magnetic resonance images (MRI) and/or computed tomography (CT) images. However, contrast media (CM) are unsuitable for certain patients with impaired renal function. Herein, we describe two limited BM cases not amenable to CM, which were treated with five-fraction (fr) SRS, without whole brain radiotherapy (WBRT), through a target definition based on non-CE-MRI. These included synchronous and partly symptomatic four BMs from esophageal squamous cell carcinoma (Case 1) and one presymptomatic regrowing lesion after WBRT for BMs from lung adenocarcinoma (Case 2). In both cases, all BMs were visualized as well-demarcated mass lesions almost distinguishable from the affected parenchyma on non-CE-MRI, particularly on T2-weighted images (WI). The gross tumor volume (GTV) was defined mainly based on T2-WI under a comprehensive comparison of non-CE-T1/T2-WIs and CT for SRS planning under image co-registration and fusion. Stereotactic radiosurgery was implemented with volumetric modulated arcs using a 5-mm leaf width multileaf collimator, for both of which 5 fr was selected, considering the maximum tumor volume and the effects from WBRT, respectively. Dose distribution was designed to ensure a moderate dose attenuation margin outside the GTV boundary and a concentrically-laminated steep dose increase inside the GTV boundary. Specifically, the peripheries of the GTV and 2 mm outside the GTV boundary were covered by ≥43 Gy with <70% isodose relative to the maximum dose and ≥31 Gy, respectively. The not-too-steep dose spillage margin can cover potentially invisible tumor invasion outside the GTV and other inherent uncertainties regarding target definition and irradiation accuracy. Post-SRS tumor responses were excellent clinically and/or radiographically with mild adverse radiation effects in Case 2. In limited BM cases unsuitable to CM, multi-fraction SRS with non-CE-MRI-based GTV definition and sufficient GTV dose along with moderate dose spillage margin would be a valuable treatment option for selected cases, with the entire GTV boundaries being almost visible on non-CE-MRI.

## Introduction

Stereotactic radiosurgery (SRS), either single- or multi-fraction (sf or mf), if appropriately designed and planned, is a highly beneficial local treatment option for non-disseminated and non-miliary brain metastases (BMs). In principle, the gross tumor volume (GTV) or clinical target volume (CTV) is defined as an enhancing lesion on contrast-enhanced (CE) magnetic resonance imaging (MRI) and/or computed tomography (CT) images [1]. Additionally, a CE-T1-weighted image (WI) is indispensable for the accurate diagnosis of central nervous system metastases, including the presence of tiny parenchymal metastases, leptomeningeal spread, and cerebrospinal fluid dissemination. When enhancing lesions on CE-T1-WI are strictly compared with those visible on T2-WI under image co-registration and fusion in the same magnification and coordinates, i.e., T1/T2 matching [2], in most cases the configuration of the enhancing lesion is almost equivalent to or slightly larger than the visible lesion on T2-WI (T2 mass lesion, T2ML) [2,3]. In the latter, the enhancing lesion may reflect the CTV, including microscopic brain invasion with a mean depth of 1 mm rather than GTV [4]. However, excessive exudation of contrast media (CM) into the surrounding brain tissue, e.g., comet-tail, or contrasting feeble and/or partial enhancement, i.e., T1/T2 mismatch, are observed in certain cases [5,6]. Considering the variability of the contrast enhancement of BM, likely susceptible to the severity of the perilesional edema, the T2ML, if clearly visualized, would reflect the true GTV more accurately than the corresponding enhancing lesion [3]. Given the attribute of contrast enhancement as indirect rather than direct visualization of the GTV/CTV, CE-T1/T2 matching has been our basis for GTV definition since 2003 [2,7,8]. When a remarkable discrepancy is observed between the enhancing lesion and the T2ML, the T2ML has been prioritized as a basis for GTV delineation since 2018 [3]. However, CM is unsuitable for certain patients with severe allergies and/or impaired renal function (IRF). To our knowledge, non-CE-CT/MRI-based target definition for sf/mf SRS of BMs has not been previously reported. Whole brain radiotherapy (WBRT) alone is preferentially given to such cases, even with single or limited BMs, despite the limited efficacy and inherent risk of acute and late detrimental effects. Image-guided 3D-conformal focal radiotherapy (to GTV) can be an alternative to WBRT while the anti-tumor efficacy is usually inferior to that of SRS with sufficient dose [9].

Since the introduction of linac-based frameless SRS in 2009 [10], we have used a T2ML as a surrogate for GTV in selected cases not amenable to CE-CT/MRI. However, modest marginal dose and internal dose heterogeneity were adopted for these cases aiming at modest and temporary local tumor control. Compromises in dose prescription and coverage often resulted in insufficient anti-tumor efficacy. Since 2018, our principles of dose prescription and its gradient near the BM boundary have been changed in order to further improve efficacy and safety [3,6]. Our strategy includes 1) ≥98% (D_98%_) of the GTV periphery covered with the dose equivalent to the biological effective dose of ≥80 Gy, based on the linear-quadratic formula with an alpha/beta ratio of 10 (BED_10_); 2) 2-3 mm outside the GTV boundary, covered with BED_10_ of approximately 50 Gy, i.e., moderate dose attenuation margin; 3) concentrically laminated steep dose increase inside the GTV boundary, i.e., very inhomogeneous GTV dose; and 4) a variable and flexible number of dose fractions with 3-15 fr according to the tumor volume, location, and proximity between tumors [3,6,11-13]. Since 2021, the SRS strategy has also been implemented with volumetric modulated arcs (VMA) using a 5-mm leaf-width multileaf collimator (MLC) [12,14]. Herein, we describe two IRF cases with limited BMs treated with SRS alone with ≥43 Gy in five-fraction (fr) through target definition based on non-CE-MRI/CT.

This report was part of the clinical study approved by the Clinical Research Review Board of Kainan Hospital Aichi Prefectural Welfare Federation of Agricultural Cooperatives (20220727-1).

## Case presentation

Case 1

**Figure 1:**
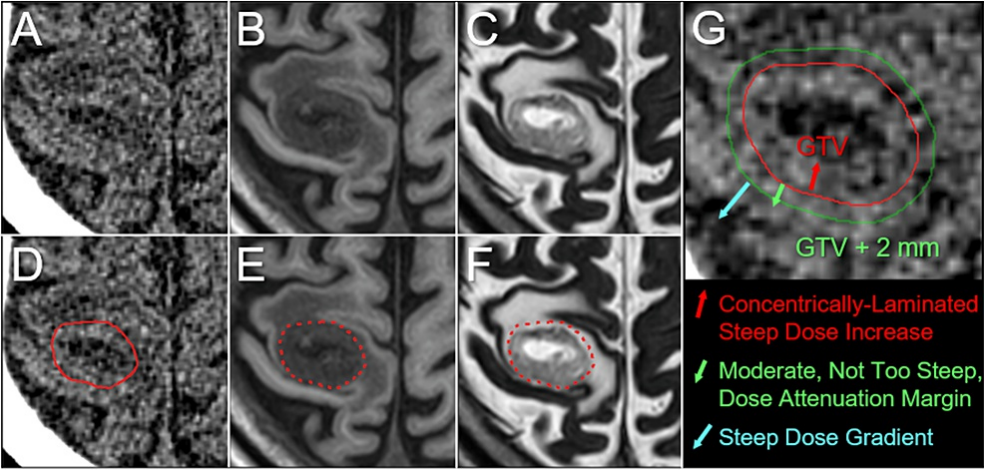
An example (Case 1) of target definition based on non-contrast-enhanced magnetic resonance images and principles of dose distribution design The images show computed tomography (CT) images (A, D, G); T1-weighted images (WI) (B, E); T2-WI (C, F); gross tumor volume (GTV) contour superimposed on the images (D-G) and GTV + 2 mm object (G); our principles of dose distribution design (G); and dose-volume histograms (DVH) (H). (A-F) All images are shown in the same magnification and coordinates under co-registration and fusion. A heterogeneously high-intensity mass lesion on T2-WI with low intensity on T1-WI is distinguishable from the brain parenchyma with or without edema. Although a visible mass on T2-WI is prioritized as a basis for GTV definition, the GTV is finally defined based on a comprehensive comparison of those visible on T2-WI, T1-WI, and CT images. The final GTV contour is slightly larger than the visible mass on T2-WI. (G) The GTV is the most prioritized planning target volume for dose prescription. The GTV + 2 mm object for dose evaluation is generated by adding a uniform 2-mm margin to the GTV.

A 72-year-old man, with a previous history of chronic kidney disease (CKD), diabetes, and cancers of the tongue and bladder, was incidentally diagnosed with locally advanced esophageal squamous cell carcinoma associated with multiple lung and liver metastases during a routine examination. The patient had mild dysphagia with the Karnofsky’s performance status (KPS) being 70%. The patient received a first line of chemotherapy with an 80% dose of the mFOLFOX6 regimen (5-fluorouracil, leucovorin, and oxaliplatin) without bevacizumab with concurrent radiotherapy (CRT) to the locoregional disease. However, the patient experienced a partial seizure followed by mild left-sided hemiparesis immediately after the initiation of chemotherapy. Non-CE-CT and MRI revealed four BMs. One of them was associated with intra-tumoral hemorrhage in the right frontal lobe, which was conservatively managed. mFOLFOX6, which was discontinued halfway, was resumed after one week in response to no enlargement of the intra-BM hemorrhage; however, due to myelosuppression, only one course was given during CRT. On the next day after the completion of CRT (1.4 months after the initiation of mFOLFOX6), which resulted in a partial response of the primary lesion, 5-fr SRS with VMA was delivered for BMs, through target definitions based on non-CE-MRI/CT. The dedicated software MIM Maestro^TM^ (Cleveland, OH: MIM Software) was used for image co-registration and object contouring [3]. Each GTV was contoured mainly on T2-WI under a comprehensive comparison of these images (Figure 1).

**Figure 2:**
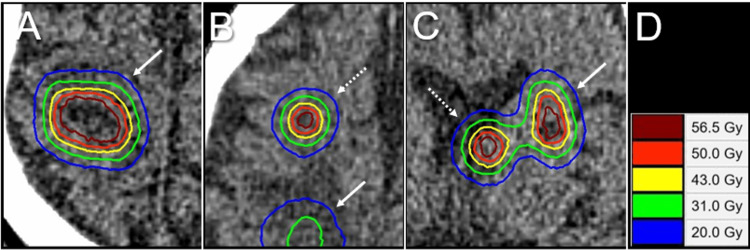
Case 1: Dose distributions for 5-fraction stereotactic radiosurgery of four brain metastases The images show (A-C) axial CT images superimposed on dose distributions for BMs in the right pre-central gyrus (arrows in A, B) (A), the right middle frontal gyrus (dashed arrow in B) (B), the left basal ganglia (arrow in C), and the left lateral ventricle (dashed arrow in C) (C); and representative isodoses (D). CT: computed tomography

**Figure 3:**
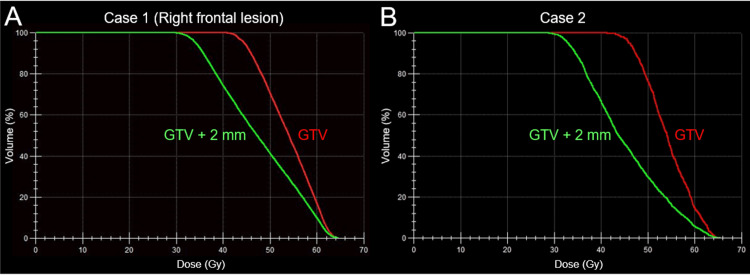
Representative dose-volume histograms for Cases 1 and 2 The images show (A, B) dose-volume histograms for the right pre-central gyrus lesion in Case 1 (A) and the left temporal lobe lesion in Case 2 (B). (A) The D_98%_ doses of the GTV and GTV + 2 mm correspond to 66.6% and 50.5% isodose, respectively, normalized to 100% at the maximum dose. Thus, a very heterogeneous GTV dose is intentionally preferred to ensure a concentrically limited steep dose increase inside the GTV boundary. GTV: gross tumor volume

The treatment platform was Agility® MLC (Elekta AB, Stockholm, Sweden) mounted in a linac Infinity® (Elekta AB) with a flattening filter-free mode of a 6 MV X-ray beam, which provides a dose rate of up to 1400 monitor units per minute [12,14]. The planning system Monaco® (Elekta AB) was used to optimize VMA for the simultaneous irradiation of four BMs via a single isocenter located in the center of the cranium. The arc arrangement consists of one coplanar arc and two non-coplanar arcs with each arc length of 360º, 180º, and 180º, respectively, which are allocated at 60º intervals to divide the cranial hemisphere evenly [12,14]. The required treatment time was within 25-30 minutes per fraction. Our principles of dose distribution for SRS were mentioned above and also described previously (Figure 1) [3,12-14]. The peripheries of the GTV and 2 mm outside the GTV boundary were covered by ≥43 Gy and ≥31 Gy, respectively, which were equivalent to BED_10_ of ≥80 Gy and ≥50 Gy, respectively (Figures 2, 3 and Table 1).

**Table 1:** Dosimetric parameters of five-fraction stereotactic radiosurgery in Cases 1 and 2 ^#^ The irradiated volume includes two lesions due to the close location. In Case 2, the intended prescription dose was 43.6 Gy (BED_10_ 81.6 Gy, single dose equivalent to 24 Gy) to ≥99% of the GTV. GTV: gross tumor volume; D_98%_: a minimum dose encompassing ≥98% of the target volume; IDS: isodose surface; D_max_: maximum dose (= D_0.001 cc_); 24 Gy vol.: an irradiated isodose volume receiving ≥24 Gy, including GTV; V_28.8 Gy_: the surrounding tissue volume outside the GTV, receiving ≥28.8 Gy; Rt: right; Lt: left

Case	Location	GTV	Maximum diameter	GTV + 2 mm		GTV			GTV – 2 mm	D_max_	24 Gy vol.	V_28.8 Gy_
				D_98%_	31 Gy coverage	D_98%_	% IDS (D_98%_)	43 Gy coverage	D_98%_			
1	Rt pre-central	6.21 cm^3^	26 mm	32.6 Gy	99.5%	43.0 Gy	66.6%	98.0%	51.0 Gy	64.6 Gy	17.58 cm^3^	7.41 cm^3^
1	Rt middle frontal	0.44 cm^3^	10 mm	32.9 Gy	99.8%	45.3 Gy	75.1%	100.0%	55.4 Gy	60.3 Gy	3.22 cm^3^	1.74 cm^3^
1	Lt basal ganglia	1.21 cm^3^	18 mm	32.4 Gy	99.4%	43.7 Gy	71.6%	98.8%	54.0 Gy	61.0 Gy	10.68 cm^3 #^	5.82 cm^3 #^
1	Lt lateral ventricle	0.72 cm^3^	11 mm	32.9 Gy	99.6%	44.7 Gy	72.3%	99.7%	53.9 Gy	61.8 Gy		
2	Lt temporal	0.71 cm^3^	11 mm	31.5 Gy	98.7%	44.9 Gy	68.7%	99.6%	58.5 Gy	65.4 Gy	3.28 cm^3^	1.70 cm^3^

**Figure 4:**
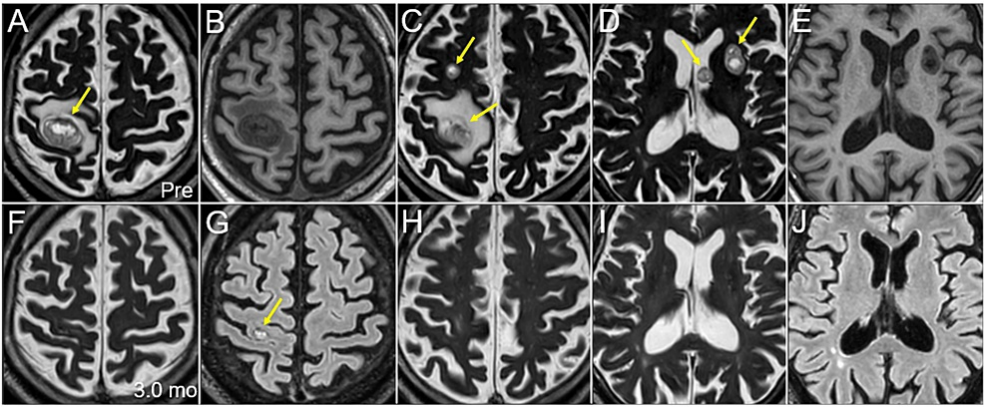
Case 1: Magnetic resonance images before and after five-fraction stereotactic radiosurgery The images show axial magnetic resonance images (MRI) (A-J); before SRS (Pre) (A-E); 3.0 months (mo) after SRS (F-J); T2-WI (A, C, D, F, H, I); T1-WI (B, E); and fluid-attenuated inversion recovery (FLAIR) images (G, J). (A-J) All images are shown in the same magnification and coordinates under co-registration and fusion. (A, C, D) A total of four mass lesions is observed (arrows in A, C, D). The intraventricular lesion probably originates from the choroid plexus in the lateral ventricle. (F-J) At three months after SRS, nearly complete remissions on T2/FLAIR images are observed in all the lesions, except for one in the right pre-central gyrus, the tumor remnant of which is only visible on the FLAIR image (arrow in G). WI: weighted image; SRS: stereotactic radiosurgery

Although the V_28.8 Gy_, the irradiated isodose volume receiving ≥28.8 Gy outside the GTV, in the motor cortex exceeded >7 cm^3^ [15], five- fraction was selected in view of the expected prognosis due to insufficiently controlled lung and liver metastases. The patient’s neurological symptoms disappeared within one month. Non-CE MRI at three months after SRS showed nearly complete remission (CR) of all the lesions along with the disappearance of pre-existing perilesional edema (Figure 4).

Post-SRS anti-cancer pharmacotherapy included one course of mFOLFOX6 (1 month after SRS) followed by nivolumab (3.3 months after SRS) in response to the progression of lung and liver metastases. Due to the declining general condition, the patient transitioned to palliative care 8 days after administration of nivolumab and died 4 months after SRS.

Case 2

**Figure 5:**
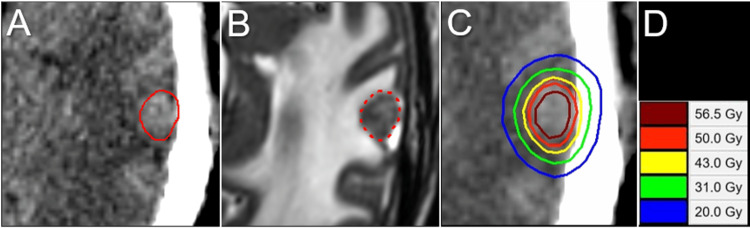
Case 2: Target definition and dose distribution for five-fraction stereotactic radiosurgery The images show planning axial CT (A, C); axial T2-WI (B); the GTV contour superimposed on the images (A, B); dose distribution (C); and representative isodoses (D). (B) A solid mass lesion mainly involved with the cortex in the left temporal lobe. CT: computed tomography; WI: weighted image; GTV: gross tumor volume

A 65-year-old woman was referred for treatment of asymptomatic BM regrowth in the left temporal lobe, 10 months after WBRT with 30 Gy/10 fr, four years after the diagnosis of metastatic lung adenocarcinoma harboring an epidermal growth factor receptor (EGFR) L858R mutation in exon 21. The patient’s past four-year anti-cancer treatments included an initial single-fr SRS for synchronous and symptomatic two BMs, a first line of erlotinib, a second line of osimertinib following verification of the EGFR T790M mutation in exon 20, a third line of carboplatin and nab-paclitaxel, WBRT in 30 Gy/10 fr for multiple metachronous BMs 37 months after initial SRS, and a fourth line of docetaxel (DTX) up to 10 courses. The KPS before salvage SRS (sSRS) was 70%, with the decline due to decreased body weight and debilitation; and the extracranial dominant active disease included a primary tumor in the left pulmonary hilum and bone metastasis in the third lumbar vertebra. Salvage SRS was initiated 17 days after the last administration of DTX and 10.4 months after WBRT. The treatment content of SRS is described in Table 1 and Figures 3, 5.

**Figure 6:**
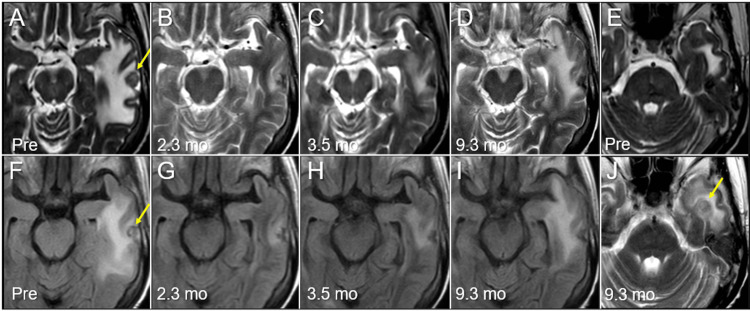
Case 2: Magnetic resonance images before and after five-fraction stereotactic radiosurgery The images show axial T2-WI (A-E, J); axial FLAIR images (F-I); before SRS (Pre) (A, E, F); at 2.3 months (mo) after SRS (B, G); at 3.5 months (C, H); and at 9.3 months (D, I, J). (A) A mass lesion (arrows in A, F) in the left temporal lobe is associated with perilesional edema. (B-D, G-I) The tumor shrinkage is observed at 2.3 months, and the low-intensity tumor remnant remained stable at 9.3 months. The perilesional edema significantly decreased at 2.3 months with a subsequent gradual increase. (J) A new lesion is observed in the left temporal base (arrow in J), which contributes partly to the aggravation of the perilesional edema in the left temporal lobe. WI: weighted image; FLAIR: fluid-attenuated inversion recovery; SRS: stereotactic radiosurgery

Re-irradiation for regrowth after WBRT requires the same prescribed dose with the same BED_10_ as the radiation-naïve case to achieve the similar anti-tumor efficacy. Therefore, the 5-fr dose equivalent to a single dose of 24 Gy, was selected as the marginal dose of the GTV to ensure efficacy and safety. After sSRS, chemotherapy was discontinued, considering the general condition. The tumor response and adverse radiographical effects after sSRS are described in Figure 6.

Systemic re-evaluation 3.8 months after sSRS in response to tumor marker elevation revealed the limited active disease with the aforementioned primary lesion and bone metastasis; therefore, stereotactic body radiotherapy was applied to both lesions 4.7 months after sSRS. A fifth line of afatinib with 20 mg/day (50% dose) was administered every other day, 6.4 months after sSRS, in response to tumor marker increase and newly developed multiple BMs (data not shown); with subsequent adjustment between consecutive days and every other day up to three months. The general condition worsened due to bacterial bronchitis at 9.2 months after sSRS, and the patient transitioned to palliative care. The patient died 11.1 months after sSRS and 58.4 months after initial SRS.

## Discussion

To the best of our knowledge, this is the first report describing efficacious and safe SRS for BMs through target definition based on non-CE-MRI, although the follow-up periods were limited. On T2-WI, the entire perimeter of the GTV (T2ML) is not necessarily distinguishable from the affected brain parenchyma, especially in cases with iso-density/intensity mass to the cerebral cortex or cerebellar parenchyma. Additionally, potential microscopic brain invasion (MBI) can fall outside the T2ML and thereby compromise anti-tumor efficacy. Pathological studies revealed that the maximum depth of MBI is 1-2 mm or more depending on the histopathological type, e.g., melanoma and small cell lung cancer, which are highly invasive, and tumor volume [4,16]. Thus, the T2MLs entail more inherent uncertainties regarding the accuracy of target definition than enhancing lesions. Therefore, a moderate, daringly not-too-steep, dose spillage margin outside the GTV boundary becomes more important to cover the potentially invisible MBI [3,6,12,13], although a steep dose gradient is originally an important characteristic of SRS [17]. We have considered that preservation of BED_10_ ≥50 Gy to at least 2 mm outside the GTV boundary, is necessary for the control of MBI even for small BMs [3,12,13]. The moderate dose attenuation margin can cover not only invisible MBI but also other inherent uncertainties regarding target definition and irradiation accuracy to ensure anti-tumor efficacy, non-inferior to WBRT. In SRS with 5 mm MLC-based VMA, dose prescription with BED_10_ ≥80 Gy to the GTV margin along with extremely inhomogeneous GTV dose tends to ensure moderate dose spillage margin with BED_10_ ≥50 Gy to the 2-3 mm outside the GTV boundary, except for small BMs [12]; whereas ill-considered dose constraint to the internal dose of GTV likely impairs dose distribution by increasing the excessive dose to the surrounding normal tissue [12,18]. Meanwhile, the very heterogeneous GTV dose, particularly the concentrically laminated steep dose increase inside the GTV boundary, can lead to early tumor shrinkage with a gradual increase in the actual minimum absorbed dose of the GTV during the irradiation period [3,19]. Sufficient dose preservation with internal dose escalation would be safer for T2ML than an enhancing lesion, as T2ML would contain almost no functioning brain tissue. In Case 1, the initial tumor responses following 5-fr SRS were excellent, despite the post-SRS chemotherapy being limited to one course of mFOLFOX6. Before 2018, we commonly adopted 35 Gy (BED_10_ 60 Gy) with a 70-80% isodose to cover the periphery of 1 mm outside the GTV boundary, which resulted in a partial response, i.e., substantial remains of viable tumor tissue, in most cases. Since 2018, as far as the other tumor responses are concerned, we consider that the current SRS strategy with marginal and internal dose escalation of the GTV along with a moderate dose spillage margin is appropriate so far. The principles would also be applicable to cases with the entire boundary of T2ML being almost visible. Although the target volume boundary, with approximately 1-3 mm margin added to the GTV, is usually a basis for dose prescription and planning in most linac facilities [18], the GTV internal dose tends to be homogeneous, with the GTV marginal dose being subject to variability [18].

Other considerations pertaining to non-CE-MRI-based planning include image distortion, optimal MR sequence, and limitations of non-CE-MRI. The potential distortion of T2-WI compared to T1-WI/CT can contribute to impaired treatment accuracy. Recently developed dedicated software, namely, Brainlab® Elements Cranial Distortion Correction (Munich, Germany: Brainlab AG) may address this concern [20]. Due to examination time constraints, planning MRI has been limited to T1/T2-WI and diffusion-weighted images. In Case 1, the maximum BM showed CR on T2-WI at three months while the tumor remnant was visualized on the corresponding FLAIR image. The significance of adding FLAIR images is an issue for future studies. Target definition based on T2ML inevitably entails the possibility of overlooking tiny parenchymal metastases and/or leptomeningeal/CSF spread. The Infinity platform with Monaco also enables the implementation of 10-fr SRS simultaneously combined with WBRT of 25-30 Gy via VMA with a simultaneous integrated boost. Therefore, for patients harboring limited BMs who can expect a long-term prognosis, SRS combined with WBRT is considered, in which the WBRT dose is adjusted according to the degree of response and feasibility expected from anti-cancer medication. This report warrants further investigation to verify the long-term efficacy, safety, and limitation of non-CE-CT/MRI-based target definition in SRS for BM.

## Conclusions

Multi-fraction SRS with non-CE-MRI-based GTV definition, along with sufficient GTV dose with a moderate dose spillage margin, would be a valuable treatment option for selected cases with limited BM, which are not amenable to CE-CT/MRI, with the entire GTV boundary being almost visible on non-CE-MRI/CT.
